# “Having One Person Tell Me I Didn’t Do the Wrong Thing”: The Impact of Support on the Post-Sexual Assault Exam Experience

**DOI:** 10.1177/10778012231156153

**Published:** 2023-02-16

**Authors:** Elizabeth Novack Wright, Sheridan Miyamoto, Jocelyn Anderson

**Affiliations:** 1 College of Nursing, 8082 The Pennsylvania State University, University Park, PA, USA; 2 The Child Maltreatment Solutions Network, 8082 The Pennsylvania State University, University Park, PA, USA

**Keywords:** sexual assault, social support, coping

## Abstract

Social support following a sexual assault (SA) may help minimize or prevent the myriad of negative sequelae impacting individuals who experience SA. Receiving a SA exam may provide initial support during the SA exam and set up individuals with needed resources and supports post-SA exam. However, the few individuals who receive a SA exam may not stay connected to resources or support post-exam. The purpose of this study was to understand individuals’ post-SA-exam social support pathways including individuals’ ability to cope, seek care, or accept support following a SA exam. Interviews were conducted with individuals who experienced SA and then received a SA exam through a telehealth model. The findings revealed the importance of social support during the SA exam and in the months that followed. Implications are discussed.

## Introduction

Approximately one in three women and one in six men in the United States experience some form of sexual violence in their lifetime, often resulting in both acute and chronic negative health sequelae (Breiding et al., 2014; Peter-Hagene & Ullman, 2018). Among individuals who experience a sexual assault (SA), 30–65% will develop lifetime post-traumatic stress disorder (PTSD), 12–40% will develop generalized anxiety, and almost 50% will experience symptoms of somatic pain (Clum et al., 2000; Pegram & Abbey, 2016; Resnick et al., 1993; Rothbaum et al., 1992; Siegel et al., 1990; Zoellner et al., 2000). However, having positive social support, both formal and informal, following a SA can help decrease mental health symptoms (Dworkin et al., 2018: Ullman & Peter-Hagene, 2016), improve coping (Orchowski et al., 2013), and provide needed validation and further assistance to individuals (Kirkner et al., 2021). Yet many individuals report negative support following a SA which may worsen symptoms such as PTSD and depression (DeCou et al., 2017), leading to cessation of further disclosure or support seeking (Ahrens, 2006; Liang et al., 2005; Richter et al., 2021; Ullman, 1996, 2007), and increase maladaptive coping mechanisms such as risky alcohol or substance misuse (McQueen et al., 2021; Ullman et al., 2018). To mitigate the harmful sequelae of trauma, it is important to understand differences in post-SA support pathways to help better connect survivors with positive support.

Receiving a SA exam from Sexual Assault Nurse Examiners (SANEs), nurses that receive specialized training in trauma-informed care and forensic evidence collection, may be an important step in connecting individuals with positive support following a SA exam. A SANE can serve as a bridge to community resources designed to promote healing and reduce the effects of trauma (Campbell et al., 2005). Individuals who received a SA exam from a SANE reported feeling believed and listened to and may be more likely to receive a complete medical exam compared to those who receive non-SANE care (Campbell et al., 2005). Those who receive care from SANEs may be more likely to have positive experiences with law enforcement and see their SA case progress through the criminal justice system compared to those receiving SA exams from untrained providers (Campbell, Patterson & Bybee, 2012). While there is literature detailing the secondary trauma that can occur for survivors of some law enforcement interactions (Lorenz et al., 2019; McQueen et al., 2021; Murphy-Oikonen et al., 2020), when there is positive support and engagement from law enforcement, it can lead to greater emotional well-being and feelings of validation amongst survivors (Greeson et al., 2014).

However, only 21% of individuals who experience a SA receive a SA exam at all and many of those are not performed by SANE examiners (Zinzow et al., 2012). Fear, stigma, and lack of knowledge about what a SA exam entails can keep SA survivors from accessing this important resource. Access to SANE led-exams is also limited as many communities across the United States, especially rural areas, have no or few SANEs available due to factors including lack of funding for specialized training and administrative support as well as strains on the nursing workforce (United States Government Accountability Office [GAO], 2016). To help combat the shortage of trained SANES, providing SA exams via telehealth with trained SANEs has been a model of care increasing across the country (Miyamoto, Thiede, Dorn et al., 2021; Miyamoto, Thiede, Wright et al., 2022; Shaw et al., 2022; Walsh et al., 2019). Telehealth models of care show promise in ensuring individuals have equitable access to trained SA providers regardless of where they live (Miyamoto, Thiede, Dorn et al., 2021; Miyamoto, Thiede, Wright et al., 2022; Shaw et al., 2022; Walsh et al., 2019). Similarly, the telehealth model may also ensure that the SANE can provide adequate discharge planning including connection with local resources and follow-up care. Telehealth allows an experienced, certified SANE to support a local SANE-trained nurse as they provide the exam. Having two nurses present means the patient may receive a more comprehensive exam. For example, one nurse may focus on evidence collection while the other nurse can focus on the psychological and support needs of the patient (Miyamoto, Thiede, Dorn et al., 2021; Miyamoto, Thiede, Wright et al., 2022).

Of those individuals who receive a SA exam, only 28–36% follow up with recommended care or resources (Ackerman et al., 2006; Darnell et al., 2015; Gilmore et al., 2018; Holmes et al., 1998). Loss to follow-up may lead individuals to cope in isolation (Ackerman et al., 2006; Campbell, 2006; Monroe et al., 2005; Ullman, 2007; Ullman et al., 2018; Ullman & Peter-Hagene, 2014; Ullman & Relyea, 2016; Walsh et al., 2012, 2013; Young-Wolff et al., 2018). Coping without support can lead to negative coping behaviors such as self-blame and denial, which are associated with greater depressive symptoms (Flicker et al., 2012). Similarly, seeking support from those who provide negative responses to a SA disclosure can lead to greater PTSD symptoms (Ullman & Peter-Hagene, 2014). Being believed and listened to when disclosing SA is associated with better adjustment in post-SA-healing (Ullman, 1996). Thus, ensuring individuals who receive a SA exam are connected to and receive support from sources that can provide therapeutic, trauma-informed responses is critical to SA healing. While research has given insight into barriers to seeking or accepting care for SA such as fear, mistrust of police, or privacy concerns, more information is needed on the experiences and pathways of support following a SA exam (Ullman & Townsend, 2007; Wolitzky-Taylor et al., 2011).

Advocates, individuals trained to provide counseling, medical and legal advocacy, and support through victim advocacy agencies, are important members of the care team during a SA exam. They also are an ideal source of support once someone leaves the hospital. Receiving support from an advocate can improve coping skills (Howard et al., 2003), reduce future risk of assault (Sullivan & Bybee, 1999), and help SA cases progress through the criminal justice system (Campbell, 2006; Patterson & Tringali, 2015). Yet, advocacy services are often underutilized. Ullman and Townsend (2007) identified multiple barriers to seeking advocacy services including lack of knowledge about resources, the stigma of receiving services, fear of experiencing bias due to race or sexual orientation and feeling unworthy of receiving services. Having an advocate present during a SA exam can be an important point of first contact and mitigate some barriers to seeking out or accepting advocacy services once the individual leaves the hospital.

### Current Study

Models of care that successfully connect individuals with resources and support during and following a SA exam and meet the individuals’ unique needs should be integrated into the initial response to trauma to facilitate early and ongoing acceptance of support. A telehealth model connecting patients to expert SANEs in real time may further help the patient feel supported during the exam and empower them to seek and engage in additional support following the exam. However, there is limited knowledge about individuals’ post-SA exam support pathways or experiences and their ability or desire to further access support (Ackerman et al., 2006; Darnell et al., 2015). The limited evidence that exists has found that social support and having a SANE perform the SA exam are associated with receiving follow-up medical care or counseling (Darnell et al., 2015) while having a mental illness, being assaulted by a partner, or drug use prior to SA is associated with a lower likelihood of receiving follow-up medical care or counseling (Ackerman et al., 2006; Darnell et al., 2015). A more nuanced understanding of different post-SA exam support pathways and factors impacting further utilization of services, coping mechanisms, and healing following an SA exam coming from the perspectives of survivors themselves is needed. This information can provide SANEs, law enforcement personnel, and advocacy professionals information to improve how responders reach survivors and engage them in services targeted at reducing trauma sequelae. Therefore, the purpose of this study was to capture the post-SA exam support pathways of individuals who experienced a SA and received telehealth-supported SA exam to understand factors that impacted their ability to cope, seek care, or accept support following a SA exam.

## Methods

### Design

This study used interpretive phenomenological (IP) analysis of in-depth, semi-structured interviews of individuals who had received a SA exam as part of a larger parent telehealth intervention study aimed to improve the quality of SA care in underserved, rural areas (Miyamoto, Thiede, Dorn et al., 2021). As part of the parent study intervention, a SA patient receives a SA exam from a local SANE-trained nurse with support from an expert telehealth SANE via telehealth (teleSANE) (Miyamoto, Thiede, Dorn et al., 2021). Typically, routine practice dictated that advocates be called to participate in every SA exam. However, due to COVID-19 restrictions, not all hospitals allowed advocates to accompany patients for SA exams during the time of the current study recruitment. If advocates were not allowed to provide in-person support, many advocates were offered to participate in the SA exam via phone. While some interview participants in this current study were connected to advocates following the SA exam, the participants did not have advocates present during the exam. The parent intervention varies from the typical standard of care in many rural areas which often consists of the SA exam performed by a trained, but not certified SANE or an untrained provider with the inconsistency of advocacy involvement (Miyamoto, Thiede, Dorn et al., 2021). Using an IP approach to facilitate data collection and analysis amplifies the voices of those who experience SA and provides first-hand knowledge of the lived experience in the months following a SA exam that can be utilized to create evidence-based, patient-centered, trauma-informed care in all phases of healing (Smith et al., 2009).

A semi-structured interview guide was developed by the first author with feedback from the second and third authors. Using an IP lens, the semi-structured interview guide had open-ended questions which allowed room for participants to tell their story in their own words (Smith et al., 2009). The guide aimed to elicit post-SA exam experiences including participant identification of early indicators of negative health sequela, participant views on seeking support, and identification of the factors that may have impacted individuals seeking or accepting post-SA support services. This study received approval from the university's Institutional Review Board.

### Recruitment

Patients who received a SA exam within the past 6 months, were at least 18 years of age, and were English-speaking were recruited from hospitals participating in the larger parent study (Miyamoto, Thiede, Dorn et al., 2021). Eligible participants who participated in the parent study and who agreed to be re-contacted were re-contacted to participate in this current study.

### Data Collection and Analysis

Interviews were conducted via audio/video conference via Zoom (Zoom Video Communications Inc, 2016) by the first author, who has experience in both qualitative interviewing and working with individuals who experience violence and SA. With participant consent, the audio of each interview was recorded for transcription. After each interview, a list of community and national support resources was sent to each participant. Demographic information including age, date of SA exam, and gender was available through the larger parent study.

The audio recordings were transcribed by a professional, HIPAA-compliant transcripion service and de-identified for analysis. The data were analyzed by all the authors. Dedoose software program (Dedoose Version 8.2.14, 2019) was used as a tool to organize, review, and code data. Following the steps of IP, authors independently read and reread transcripts while noting observations and initial concepts within the transcripts (Smith et al., 2009). Using these observational notes, the authors developed clusters of meaning and themes within each transcript and then came together to discuss at regular meetings (Alase, 2017). Following discussions of the emerging themes within each interview, the research team identified salient themes substantiated by the data and drew connections across themes and interviews (Smith et al., 2009). The overall findings and themes from the interviews were categorized based on the participants’ post-SA pathways. To ensure rigor and reflexivity, peer critique and discussion occurred throughout each stage of the research process. Bracketing, the process in which the researcher makes their assumptions explicit to reduce bias in analysis, occurred prior to analyzing each interview (Alase, 2017). Consistent with IP methods, small sample sizes are recommended among a homogenous sample pool to allow for an in-depth analysis of a specific experience (Smith et al., 2009).

### Reflexivity Statement

The authors are all nurse researchers with backgrounds in trauma, violence, SA, and forensic nursing. Two have practiced as SANEs, and the third has worked as a victim advocate. In addition to nursing, we bring a strong feminist theory and research background to our work.

## Results

A total of 27 potentially eligible individuals were seen for SA exams at participating hospitals between August 2020 and April 2021. Each potential participant was contacted by the lead author regarding their interest in this secondary study. Five (19%) were contacted and declined, 17 (63%) were unable to be contacted or did not respond (e.g., did not reply to email/voicemail or phone numbers disconnected), and 5 (19%) were contacted and agreed to participate. The time between the SA exam and the interview ranged from two to five months with an average of 3 months between SA exam and study interview. Participant ages ranged from 21 to 59 years with a mean of 38 years old. The sample of participants who completed interviews was made up of cisgender women, four White and one African American. Participants received their SA exams in either rural (*n*  =  2) or suburban hospitals (*n*  =  3). All participants received their SA exam from a SANE-trained nurse and an expert SANE participating through telehealth as part of a larger intervention on improving the quality and accessibility of SA exams (Miyamoto, Thiede, Dorn et al., 2021).

When analyzing the five participants’ interviews, four distinct common elements that impacted post-assault healing and help-seeking pathways were identified. The four post-assault pathways were categorized as (a) SA exam support validates survivor experience and instills confidence to seek post-exam support; (b) Post-exam support positively impacts survivors’ ability to navigate external instability; (c) Sustained support helps individuals find meaning and purpose; and (d) Negative reactions and lack of social support leads to loss of trust and hinders disclosure.

### SA Exam Support Validates Survivor Experience and Instills Confidence to Seek Post-Exam Support

#### Providing Reassurance and Alleviating Guilt

The SA exam as a validating, positive experience was a salient and universal theme throughout all five participant interviews. Participants explained they sought SA exams because it was what they felt they needed to do, partly to seek care and validation for what they experienced. The SA exam was often seen as the first step in the recovery process where they were heard and cared for. The women described how the SA exam helped instill a sense of reassurance that the assault was not their fault and alleviated negative feelings participants had about themselves regarding their assault. The reassurance and removal of guilt often helped participants to continue to process the assault, seek further help, or seek justice. The SA exam provided the formal validation participants desired. As a participant explained, “I think it's the support network, ‘cause I finally have somebody who believes me that is not family” (Participant [P]1).

A participant who had a poor experience with law enforcement on the way to the SA exam felt the nurses provided needed reassurance to balance that initial negative interaction:

They [SANEs] were asking me how I was and completely made me feel like it was not my fault. I think the key component that I took away from [the assault] was that I was feeling dirty. I was feeling ashamed. I was feeling guilty. When I left, I wasn’t feeling those as strongly because they kept reassuring me throughout the process. I think that was imperative. Them reassuring a victim that it's not their fault, that nothing they did provoked the situation. Trying to help with those feelings of guilt and shame and just feeling gross. They really helped just to reassure me. (P3)

#### Enabling Empowerment to Seek Justice

The SA exam experience also helped some women have the confidence and desire to interact with the criminal justice system. As survivors felt believed and validated during the exam, some felt empowered to seek justice. Seeking justice outcomes and working with investigators to do so was a form of support for those who sought that outcome. Through the instilled confidence, participants saw the opportunity to work with the criminal justice system to provide safety and closure. A participant described how the SA exam gave her hope that her assailant would be arrested:

I had hope. I had hope that—I’d never been assaulted before. I’ve never gone through this, anything like this before in my life, but I had hope that when she [SANE] told me I could get dressed and walk out into the waiting area…I thought everything's gonna be okay. They’re gonna work on this. They’re [law enforcement] gonna have [assailant] arrested. I won’t have to worry anymore. (P4)

Similarly, the SA exam provided another participant the confidence to testify at trial:

I wouldn’t probably have been prepared to do so [testify] if they [SANEs] hadn’t handled me with kid gloves at hospital. If I hadn’t had that reassurance, I might have still been carrying that guilt and that shame with me. I maybe wouldn’t have been as effective on the stand. (P3)

### Post-Exam Support Positively Impacts Survivors’ Ability to Navigate External Instability *Support Helps Mitigate Instability*

All participants had to deal with additional stress or challenges outside of the SA, but their ability to overcome these external stressors depended greatly on the type of support they received from others. When participants had sustained, reliable support they were able to deal with both the external stress and manage their assault-related needs. A participant explained how her relationship with the assailant was an added stress she had to navigate following the SA:

I moved out of the situation I was living in, and I took my child with me. Then just having to still make time to—how I was going to split custody with the person that did that to me because we had a child together, so just the stress of that. (P2)

However, due to her continued interaction with community advocacy, she was provided with temporary shelter, legal support, and counseling which helped alleviate stress and help her cope.

#### Lack of Support Increases Instability

Participants who lacked post-assault support had a more difficult time overcoming external stressors. The combination of negative help-seeking experiences, lack of positive support, and the inability to manage the external stressors made it hard to access or stay connected to resources. A participant who did not have family support described the multiple job stressors and challenges she faced which made it hard for her to take time off to heal and recover from the SA:

It's been a rollercoaster….I’ve changed employment twice since then [the assault], once because my employer, even though I told him I had something going on but because we had to meet certain deadlines, they wouldn’t give it [time off] to me. That really left a sour taste in my mouth. I didn’t have time. I threw myself into another job right after that because of being a single mom. It just didn’t work out. (P1)

Even when participants were connected to support, having additional stress or instability made it hard to access resources. A participant described how she was unable to pay her phone bill meaning she was unable to continue with her phone counseling sessions with the advocate. Since then, she had been unable to recontact the advocate:

I didn’t have the money to keep the phone and things like that have happened, so I didn’t talk to her [advocate] for two weeks now. It's no fault of hers. It's all on me, and I get that. I understand that. It was nice to talk to her. It was nice to just—but she would tell me that she was gonna call me after my hearing and things like that, but she never did. I did have my phone at that point, but maybe she was just busy. (P5)

The SA and related aftermath exacerbated prior mental health issues for some participants making it challenging to focus on recovery from the SA. This same participant explained how she had trouble accessing mental health resources due to COVID-19 restrictions. Without mental help and support, she felt even more alone, “We can’t even go anywhere anymore. Us people with mental illness are feeling really alone, a lot of us are” (P5).

### Sustained Support Helps Survivors Find Meaning and Purpose

Participants who received validation and support from either family or advocacy following their SA exams showed resilience through their healing process and began to gain perspective about their SA experiences. Even though these participants each faced invalidation or lack of support from either informal or formal sources at some point in their process, having some effective support helped counteract the other negative experiences. Because of their positive support experiences of feeling validated and believed, this group of participants described their desire to help others who experience SA.

#### Reliable Support Compensates for Negative Experiences

Sustained support from family and friends following a SA exam was an important source of validation even in the absence of formal support from advocacy or mental health professionals. A participant who lacked a connection to an advocacy service after her SA exam described how important it was to have continued support from family:

That was probably one of the saving graces to know that people that know me so well were like, “Okay, what happened? Not your fault….you’re going to be strong. You’re going to just tell your truth.” There was no doubt in anyone's mind that the [assault] had happened to me. I was afraid that people wouldn’t believe me or thought that I’d made it up or—these things just don’t—you don’t know until it happens to you. Just to have them [family] validate me and my feelings, and support me through the process and go with me to the pretrial to cheer me on. (P3)

Inversely, when faced with a lack of familial support, sustained advocacy support proved to be an invaluable resource. One participant who did not have support from family, but had an ongoing relationship with advocacy explained how important advocacy can be:

After the whole thing [assault] happened she [victim advocate] just—just that phone call really made a difference for me because it made me feel like I wasn’t alone and that people actually do care what happens to women.

[Advocacy] is a big support for a woman that something like that [sexual assault] happens to …it [advocacy] takes you through feelings bundled up. The people are so kind…they let you know it’s okay…. It’s like a safety net. (P2)

#### Evolving from Trauma

Participants who experienced sustained support were beginning to gain perspective from their experiences. These participants described a process through which their self-perception evolved to include “survivor” and to share that identity with others to act as a support or source of hope.

Hopefully, if any other youth are in this or any other women are in this situation and ever needed someone to come to, I could say, ‘From personal experience, I can see what you’re going through.’ …I just wanna be a beacon of hope for other people. … Any way I can move forward and just be a strong person, set a proper example, be a role model, that's really what's important to me. That's what makes me happy as a person. (P3)

I am a musician, so I play music, and I’m actually writing a song about what happened to me and my experiences through it and how I got over it, to let people know that it's okay. They’re not alone. (P2)

### Negative Reactions and Lack of Support Lead to Loss of Trust and Hinders Further Disclosure

Multiple instances of negative interactions with informal or formal supports after the SA exam impacted participants’ further disclosure or ongoing processing with others about the assault, engagement with services, or seeking additional formal or informal support. The work done during the SA exam to instill control and make them feel believed was sometimes negated by the responses they received after they left the hospital. Even though these participants all had at least one source of support, if they experienced consistent invalidation of their experience, it led participants to feel alone and regret disclosing their SA.

#### Negative Responses from Family

Regardless of other types of support or validation, the desire to receive validation from family was extremely important to participants. Yet, for participants who did not receive that desired response, it impacted their ability to progress in their post-exam healing trajectories. Participants with this experience dealt with the SA alone, without support. A participant had her family take the assailant's (her ex-partner) side, isolating her, “ My family turned my back on me with all of this…My family's taking his side” (P1).

Similarly, another participant, who had a strained relationship with her family, had no family members reach out after the SA. Without family support, she felt that no one cared about her:

I have no support at all. None. I didn’t get one single phone call from them [family] to ask me all week how I was doing. I could have been dead, and they wouldn’t have cared. They wouldn’t have ever known. (P4)

Without additional validation, the lack of support from family or friends made it challenging for some to continue to reach out for help:

I tried to tell my daughters about it, and they both pretty much disowned me. They wouldn’t even talk to me about it. My grandchild, who I was really, really close with, I don’t see her much anymore. It was like I’m not the victim. That's how my kids look at it, and it's hurtful…. Because of that assault I ended up losing my family, my home, everything I had because of that assault, and no one believes me. I won’t even tell anyone anymore. (P5)

#### Invalidation from Law Enforcement

Poor responses from law enforcement often negated any prior reassurance participants had received about their SA experiences. The lack of support from this formal system, ideally designed to protect survivors, made it very challenging for them to feel as if the SA they experienced was a “real rape.” After feeling hope after SA exam, a participant talked about how the negative interaction with a law enforcement officer ruined the positive experience she had during the SA exam:

Then I walked out into the waiting room, and that detective was sitting there, and she looked at me, and she said, “What am I doing here?” All the good work that the nurse had done was just—all I could remember was the detective…. Any kind of hope that I had from the nurse that did the exam, any hope I had from them was totally blown away as soon as I walked out to that detective. (P5)

Another participant, whose assailant was an ex-partner, had a similar experience with law enforcement:

The cops need better training, especially in rural areas dealing with this stuff because one of the things that the cops said to me, and this has stuck with me, is that, “Are you sure you’re just not trying to get him back?” That stuck with me, because that's his exact phrase, “Are you sure you’re not trying to get him back?,” because they knew that we were fighting because of how he was treating me and stuff like that. And he's like, “Are you sure you’re just not trying to get him back and get him in trouble?” That has stuck with me. (P1)

#### Lack of Access to Advocates

While advocacy can be an important source of support for those who experience a SA, some participants did not receive follow-up from an advocate. The lack of advocacy follow-up denied them of a formal connection point to services, especially when they had no other support. A participant discussed how she never heard back from advocacy following her SA exam and how having that connection would have been beneficial:

One of the things that would have been really helpful is if the advocate would have followed through and contacted me because the advocate kind of—I saw her at the hospital, and then she just disappeared. I never heard from her again. (P1)

#### The Importance of Having at Least One Support

Even though some participants had multiple experiences of lack of support in their post-SA exam trajectories, they all had at least one source of support they relied upon. This single support was at times lifesaving. A participant described how her brief connection with advocacy saved her life when she had no one else:

I probably would have killed myself at that point [when no one would listen]. I probably would have. I mean I thought of how I was going to do it and everything. If I wouldn’t have had [advocate] to talk to at that point—that one, just one person to listen to my story and tell me I didn’t do the wrong thing. (P5)

Another participant was able to find support from a friend who had a similar SA experience with the same assailant. She also described this support as live-saving:

I probably would have committed suicide [without friend support]. I probably would’ve, or just did something just to have people to talk to. That's pretty much probably what I would’ve just done it for [attempted suicide]. It was good [to talk to a friend]. (P4)

Despite her negative support experiences from family and law enforcement, one participant was able to find support from her current partner, “In all honesty, if it wasn’t for my boyfriend and his support, I don’t honestly think I would—I think I would be not where I’m at right now. He is a huge support” (P1).

#### Loss of Trust and Unwillingness to Disclose

After multiple experiences of invalidation or lack of support, participants began to lose trust in others and the systems designed to help them. After her family turned her back on her, a participant described why she won’t reach out to anyone in the future, “I do not trust people anymore. I don’t reach out like I probably could and should because I don’t know if they’re going to screw me over” (P1).

The lack of validation one participant received made her want to no longer disclose the SA, “I wish I could’ve forgot about the rape and just never talk about it ever again. Because nobody believes me anyway” (P4).

Similarly, another participant decided she would no longer seek help or disclose the SA because no one believed her, “I would never ever, ever go back and tell anyone else because when I tell somebody, I end up—I end up being the perp, not the victim, again and again and again and again” (P5).

## Discussion

The findings in this study highlight the impact of the initial and ongoing response to SA disclosure and how that response impacts survivors’ abilities to access and receive support to begin healing from the trauma. The participants in this study described the telehealth-supported SA exam as an important step in healing by providing validation of their experience and instilling confidence in them to move forward following their SA. However, those who experience a SA cannot always control how others react to their disclosure or help-seeking. Once participants left the exam room, pathways differed depending on the responses they received from others. Sustained positive support helped participants feel confident enough to engage with the criminal justice system and begin to evolve from their experiences. Alternatively, continued negative support and invalidation of their experience hindered further help-seeking and perpetuate feelings of isolation and loss of trust in others. Our findings also emphasize how external instability outside of the SA such as employment, financial issues, and mental health can impact one's ability to successfully connect to resources after a SA.

### Importance of the Sexual Assault Exam

The initial healthcare response during the aftermath of SA can have profound impacts on the survivor (Campbell et al., 2001, 2005; Caswell et al., 2019; Kirkner et al., 2021; Maier, 2008). SANEs receive specialized training and knowledge on trauma-informed, person-centered care, and the physical and psychological impacts SA can have on the individual. This training helps SANEs provide the needed physical care and psychological support survivors deserve. The telehealth model aims to ensure access to comprehensive post-SA care while developing the expertise of local nurses (Miyamoto, Thiede, Dorn et al., 2021).

The participants in this study had positive and affirming experiences with the SANE-trained nurses. Participants further emphasized the importance of the nurses’ empathy and validation in restoring confidence and assurance. Campbell et al.’s (2005) review found that SA patients who receive care from SANEs feel believed, in control, and informed while SA patients who receive care from untrained providers may experience secondary victimization and further invalidation (Littel, 2001). In the current study participants also had the benefit of having a teleSANE help perform the exam through a telehealth platform. Thus, these participants were able to receive standardized, evidence-based care from two nurses, helping to meet all patient needs. For example, while the local nurse attends to evidence collection or the many focused tasks of conducting the exam, the teleSANE can focus on patient well-being and emotional needs. Additionally, the teleSANE can model how to provide effective support and quality care learned through their vast experience in the provision of SA care.

All participants had positive experiences during their SA exam and voiced feelings believed and listened to. Providing opportunities for individuals who experience a SA to receive supportive reactions following a SA disclosure is critical as feeling validated and supported following disclosure is associated with better mental health outcomes (Ullman, 1996). Interpersonal support from the nurse and advocacy interaction during the SA exam often gave participants the confidence they needed to continue with the criminal justice system. Even though studies have highlighted the benefits of receiving a SA exam by a SANE, such as feeling believed, receiving more medical care, having more thorough evidence collection, and having more engagement with the criminal justice system (Campbell, Bybee et al., 2012; Campbell et al., 2005), many individuals still do not seek SA exams following an experience of SA due to fear, barriers to access, or lack of understanding about SA exams (Fisher et al., 2003; Zinzow et al., 2012).

These study participants were able to seek help following SA through a SA exam. Yet there may be individuals who desire help but face barriers to seeking out a SA exam. This may be due to lack of availability of quality, specialized SA care due to a shortage of trained SANEs throughout the country (GAO, 2016; Thiede & Miyamoto, 2021). Some individuals may also forgo a SA exam due to the misconception that law enforcement must be involved. Patient-centered and trauma-informed care are the foundations of SANE-led SA exams, and the patient may receive an SA exam without the involvement of law enforcement. Telehealth models of care are designed to address these barriers and ensure all people have access to this essential specialty care (Miyamoto, Thiede, Dorn et al., 2021; Walsh et al., 2019). Furthermore, public health campaigns to destigmatize sexual assault and post-assault care, including the choice to involve or not involve law enforcement, may increase individuals’ willingness to access healthcare as the first step in their path validation, assurance, and healing.

### Impact of External Instability

While friends and family are more likely to be the recipients of SA disclosures, everyone in our sample disclosed to some formal system as evidenced by seeking a SA exam. Participants often voiced that they chose to seek out a SA exam to find closure, seek validation, and because it was seen as “something they should do.” However, some participants lost contact with formal systems or could not connect to formal systems following the exam. Other studies have found that follow-up care with medical or mental health services after a SA exam is often low (Ackerman et al., 2006; Darnell et al., 2015). Our findings offer reasons why access to follow-up services can be challenging for survivors. All participants in our study wanted help and initially sought it. Aside from the help-seeking responses, many faced external stressors that impeded their ability to seek help and heal. When participants did not have continued support, it was challenging for them to try to manage both the SA and other stressors. Those who were experiencing additional stressors such as financial issues or loss of employment had to prioritize stabilizing the additional stressors, often alone, before they could focus on the SA recovery process. Understanding and identifying potential external stressors or instabilities among those who have experienced a SA at the point of the SA exam may help decrease survivor burden and help survivors stay connected to supportive resources.

### Impact of Continued Support

Our findings revealed that continued support and validation following a SA exam has a positive, healing impact on individuals. Even among participants who had some negative experiences, having even one positive support from another source helped counteract their negative experiences. The participants who had sustained support throughout their post-exam experiences continued with the criminal justice system and began to evolve from their experiences. Previous SA literature has found positive support can promote future help-seeking and positive mental health outcomes following SA (Liang et al., 2005; Richter et al., 2021; Ullman, 1996, 2007). In this study, family and advocacy served as important support systems. Feeling supported and believed by family helped participants move forward after their SA exam. Literature has found that the response from family members can have a strong impact on mental health and recovery after SA (Ahrens, 2006). Having those closest to you believe and validate your experiences can be extremely powerful. Similarly, advocates also proved beneficial and are an important part of the care team during and after a SA exam (Campbell, 2006).

### Impact of Negative Support

The participants who did not experience sustained support, but rather had multiple negative responses from others had a more difficult time finding validation and eventually lost the desire to further disclose their assault. Lack of access to supportive resources can hinder further help-seeking (Liang et al., 2005) and is associated with more psychological symptoms and less control over recovery (Ullman, 1996). Participants who had continued negative reactions, especially from family, following the SA exam had more instances of mental health exacerbations and felt they could no longer trust others. While family and friend support can be beneficial, not being believed by family and friends can increase depressive symptoms, further exacerbate the negative impact of SA (Kaukinen & Demaris, 2009), and increase feelings of blame (Ahrens, 2006). Negative reactions from those who we trust the most can have the most devastating impact.

Participants described the invalidation they received from law enforcement and the hindrance law enforcement had on participants’ healing trajectories. Studies have documented that interactions with law enforcement can be a source of invalidation and blame following a SA (Ahrens, 2006; Campbell, 2006; Maier, 2008) Experiencing invalidation from a system that is designed to protect can have strong implications for thwarting future help-seeking as negative reactions from formal supports can lead individuals to believe further disclosure will not be effective (Ahrens, 2006). Descriptions of negative reactions after an initial positive one during the examination further emphasize the need for individuals to have access to some form of validation following the SA to support and encourage the individuals even when law enforcement does otherwise. Even though some participants in this study did not receive the desired connection or follow-up from advocacy, when advocates are present during a SA exam, patients report having more positive experiences with law enforcement (Campbell, 2006). The absence of advocates in some of the participants’ pathways missed opportunities for connections to resources or support. It would be important to understand why lack of connection to advocacy occurred. A more nuanced understanding of how victim advocacy agencies follow up with patients following a SA exam is needed.

### Implications and Recommendations

Participants in this study discussed that the support they received during their telehealth-enabled SA examination helped empower them to have the confidence to further seek follow-up support, including support from law enforcement. Empowerment and regaining control and choice are invaluable in the healing process. In some instances, participants in this study had difficulty connecting with advocacy. In low-resource settings, where advocacy during the SA exam may be limited or inaccessible, having SANEs in person or via telehealth may ensure survivors receive the important advocacy information and instructions for advocacy follow-up. In circumstances where advocates are not available or when hospital policies restrict the presence of support persons (such as during COVID-19 case surges), telehealth models to ensure remote advocacy presence should be explored when advocacy options are limited.

Our findings emphasize that individuals can lose connection to resources following a SA exam despite the structures and support advocacy and health care agencies try to implement. We must think of different ways we can help individuals who have experienced SA receive and sustain the help they desire.

Many participants in this study received negative responses or lack of support after their SA exam. To better prepare individuals once they leave the hospital, SANEs and the SA care team must advocate for their patients both during and after the exam. Nurse-advocacy partnerships that begin during the SA exam and extend once the patient is discharged can better ensure SA patients are given the opportunity for follow-up and support from both nurses and advocates. Providing anticipatory guidance on what to expect upon leaving the hospital such as possible responses from family or friends, potential interactions with law enforcement, and when to expect or request follow-up contact from both the SANE and advocate can help prepare individuals on how to navigate their post-exam needs. Pre-discharge needs assessments should also be conducted to develop a plan that is tailored to address patients’ potential barriers to follow-up.

Education for family during and after the SA exam can allow for family to understand the importance of familial support and response to disclosure. Providing the patient with the option to include a support person during certain aspects of the SA exam, such as discharge, may give the family member or friend the opportunity to see formal sources (i.e., SANE) respond to the SA and validate the experience, which could be an important teachable moment. Providing outreach to family or friends following the SA exam with a nurse-advocacy partnership through group, family, or individual counseling or educational sessions can also teach informal support about how to respond to disclosures and how their reactions have lasting implications on their loved one's well-being. Further training for law enforcement on appropriate responses to sexual assault and their impact on the help-seeking trajectory of individuals is needed.

### Strengths and Limitations

This study is strengthened by the in-depth and personal reflections of individuals who have experienced a SA and SA exam, a group known to be challenging to engage (Btoush, & Campbell, 2009). The participants shared difficult and traumatizing experiences with the hope that they may be able to help others. Our study has several limitations. Interpretative phenomenological allows for small sample sizes to gain in-depth understandings from homogenous samples. Our findings may have transferability to primarily white cisgender women; making it difficult to generalize findings or examine perspectives of other populations. Recruitment of participants who have experienced sexual violence has historically been difficult due to safety, ethical, or logistical reasons (Btoush, & Campbell, 2009). Those who declined to participate in this study may have had different experiences with support following their SA exams leading to reluctance in speaking further about their experience with the research team. Additionally, recruitment of participants occurred during the COVID-19 pandemic, which saw a drop in individuals seeking SA exams (Wright et al., 2022), furthering recruitment challenges. Yet the depth of the interviews provided important insights adding to our understanding of the post-SA exam pathways and the participants in this study were eager to share their experiences. They often expressed it was “helpful” for them to speak with the research team as another outlet to share what happened to them while receiving empathy and kindness. While it can be difficult to reach patients after their SA exam, offering the opportunity for patients to participate in research about their assault and post-assault experience can be positive and therapeutic. Lastly, our methods allowed for a rich, nuanced understanding of each participant's journey.

## Conclusion

Ensuring individuals who experience SA are connected to supportive and helpful resources can reduce and prevent the short and long terms negative outcomes associated with SA. SA exams can be an important part of an individual's recovery from SA, yet interactions with supports, such as family or law enforcement, outside of the SA exam may further encourage or hinder help-seeking and healing. Understanding the individual's external stressors and unique barriers to follow-up after a SA exam may improve connection to important and helpful resources.
